# Health-related quality of life in long-term differentiated thyroid cancer survivors: A cross-sectional Tunisian-based study

**DOI:** 10.3389/fendo.2022.999331

**Published:** 2022-08-24

**Authors:** Abdel Mouhaymen Missaoui, Fatma Hamza, Mohamed Maaloul, Hana Charfi, Wiem Ghrissi, Mohamed Abid, Fadhel Guermazi

**Affiliations:** ^1^ Department of Endocrinology and Diabetology, Hedi Chaker Univsersity Hospital, Sfax, Tunisia; ^2^ Department of Nuclear Medicine, Habib Bouguiba University Hospital, Sfax, Tunisia

**Keywords:** thyroid cancer, health-related quality of life, cancer survivorship, psychosocial oncology, head and neck neoplasms, developing countries

## Abstract

**Background and Aim:**

The incidence of differentiated thyroid cancer (DTC) has risen dramatically worldwide. Despite an excellent prognosis, the growing DTC survivors’ community often features poor health-related quality of life (HRQoL), which challenges long-term DTC care, particularly in developing Southern Mediterranean and African countries. We aimed to assess the HRQoL and to investigate its determinants in disease-free Tunisian DTC survivors.

**Methods:**

We conducted a three-month cross-sectional study that included 266 patients diagnosed with DTC. We assessed the HRQoL in eligible participants using the short form–36 health survey, in comparison with 76 healthy controls.

**Results:**

The 86 eligible DTC survivors were predominantly female (89.5%) with an average age of 44.3 ± 12.5 years. Physical-functioning (PF), role-physical (RP), and pain domains were substantially altered compared to the reference population. Age was negatively associated with PF, RP, role-emotional (RE), and social functioning (SF). Tumor size and lymph node metastases affected general health and PF, respectively. The cancer-free survival duration was positively correlated with mental health (MH). Poor neck scar healing and persistent post-operative hypoparathyroidism significantly deteriorate MH. Pain perception was positively correlated with the radioactive iodine cumulative dose. Subclinical hyperthyroidism significantly reduced PF and RP scores. TSH suppression was negatively and strongly correlated with MH and SF scores.

**Conclusion:**

HRQoL is substantially reduced in DTC survivors compared to the normative Tunisian population. These results could be extrapolated to similar individuals in other South Mediterranean and African countries. The development of coordinated multidisciplinary aftercare interventions in this region is warranted to preserve HRQoL in DTC survivors.

## Introduction

The incidence of differentiated thyroid cancer (DTC) has risen dramatically in developed countries and internationally over the past three decades. This tendency is reported on every continent except Africa, where the diagnosis is still insufficient (1). Papillary thyroid carcinoma, especially microcarcinomas, accounts for more than 80% of all newly diagnosed cases (2). The high disparities in thyroid cancer incidence by geographic area and ethnicity are attributable to genetic predisposition, environmental factors, and easier access to medical care (3, 4). The management of DTC has been incrementally consensual. It typically consists of surgery, radioactive iodine (RAI) ablation, and TSH suppressive therapy with an excellent structural and biochemical response for 90% of patients (5–7). In the case of progressive, RAI-refractory, or metastatic disease, novel therapeutic approaches based on inhibitors of tyrosine kinases and mitotic kinases are showing promising results in preclinical and clinical trials (5, 8, 9).

A large DTC survivors’ community has been growing so far owing to its excellent prognosis and stable mortality rates, mainly in those with papillary thyroid carcinoma (10–12). Worldwide, the follow-up of DTC survivors is no longer restrained to cancer control. Similar to other chronic diseases, it involves more extensive healthcare aspects such as metabolic, cardiovascular, and psychological issues (13).

Health-related quality of life (HRQoL) is a multidimensional concept that embraces patients’ perceptions of their physical, psychological, social, and spiritual functions. Preserving HRQoL is a central target in modern cancer care (14). DTC survivors deal with subjective sequelae following diagnosis, psychological distress during treatment, and possible side effects at follow-up. All these circumstances may affect their HRQoL.

Previously published papers reported deceased HRQoL in DTC patients. The majority of them focused on the population originating from developed Western and Asian countries (15, 16). There is a lack of precise data on HRQoL in DTC survivors living in developing southern Mediterranean and African countries. More research on HRQoL in patients living in these regions should be conducted to enable local healthcare givers to offer individualized care for DTC-cured patients in accordance with their geographic, ethnic, and economic status in this region.

The objective of this study is to compare the HRQoL for disease-free survivors of DTC in Tunisia, a developing North African country, with that of the general local population. We also aim to investigate the influence of sociodemographic, clinical, and treatment procedures of DTC on HRQoL in the disease-free Tunisian community.

## Methods

### Study design

We conducted a cross-sectional study at the Nuclear Medicine Department of Habib Bourguiba University Center, Sfax, southern Tunisia. It included all disease-free patients diagnosed with DTC who attended the outpatient clinic for routine follow-up from September 1^st^ to November 30^th^, 2021.

### DTC survivors group

All recruited patients had undergone the conventional treatment protocol proposed by our center: total thyroidectomy (TT) with prophylactic central neck lymphadenectomy (pCNL), radioiodine remnant ablation (RAI), and long-term TSH suppression therapy. In our center, we follow a locally adapted version of the 2015 American Thyroid Association (ATA) management guidelines for adult patients with DTC (5). Thus, the initial TSH suppression is proposed for at least 2 years in patients within the low or intermediate ATA risk groups, and 5 years in those within the high-risk category. In the case of structural incomplete response (persistent or newly detected loco-regional or distant metastases), biochemical incomplete response (abnormal thyroglobulin or increased anti-thyroglobulin antibody levels with no localizable disease), or indeterminate response, the hormonal suppression therapy is prolonged.

**Figure 1:**
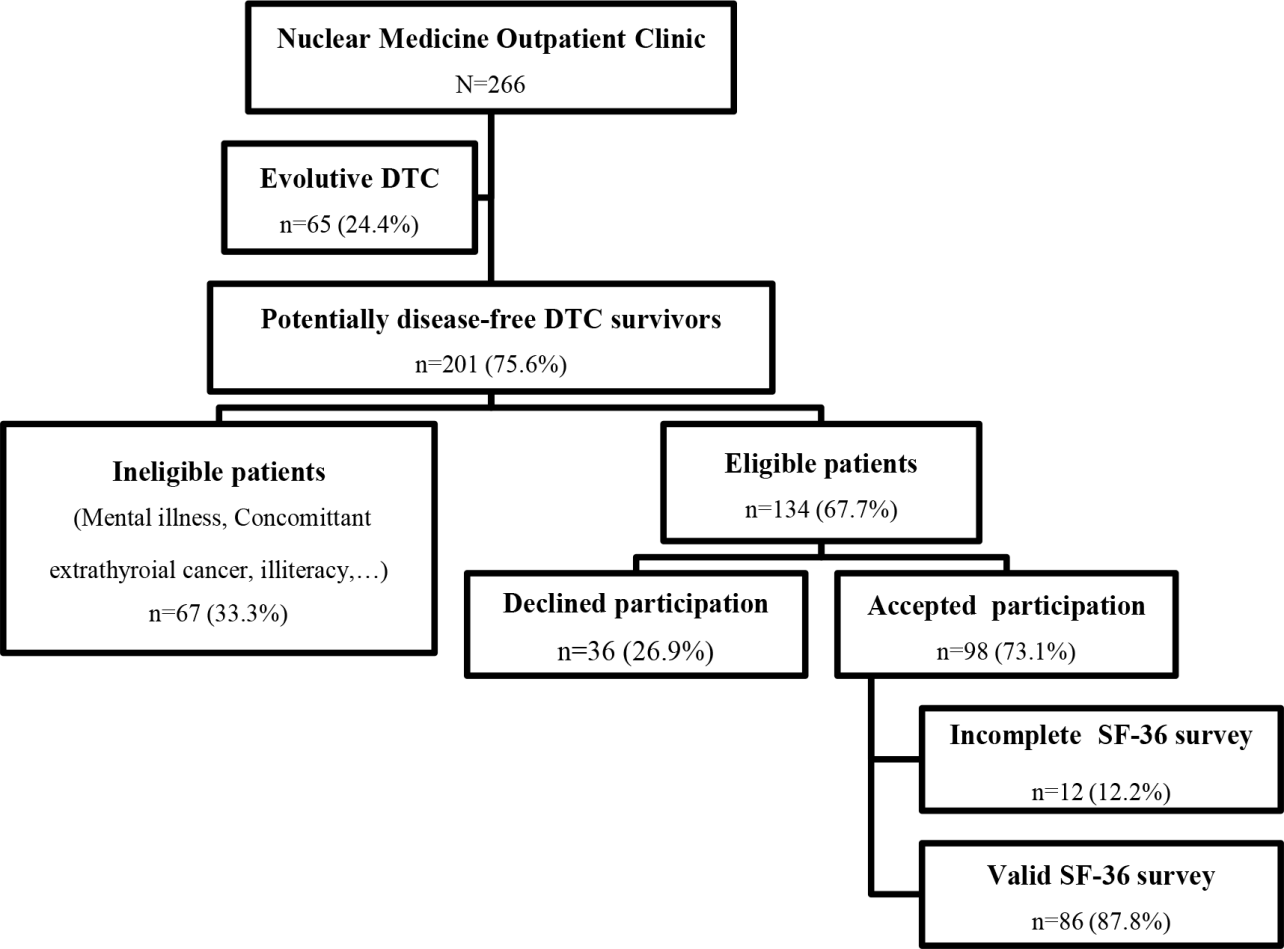
Recruitment Procedure of Eligible DTC Survivors at The Nuclear Medicine Outpatient Clinic. DTC, Differentiated thyroid cancer; SF-36, Short form 36 health survey.

The included population has to achieve at least 6-month disease-free survivorship. Ongoing RAI sessions, detectable thyroglobulin levels during TSH suppression, and non-adherence to levothyroxine replacement were the exclusion criteria. We also excluded illiterate participants and those presenting with mental illness (depression, psychotic disorders, etc.) or cognitive impairment. The inclusion and exclusion criteria are indicated in **Figure 1**.

Demographic data and socioeconomic status data were derived from a direct interview with the selected participants. Disease characteristics include disease type, histological findings, AJCC/TNM-staging (8th edition) (17), and American Thyroid Association Risk Stratification category (5). Data regarding treatment consisted of surgery outcomes, method of RAI preparation, cumulative RAI dose, and treatment side effects were derived from medical charts.

### Control group

The reference control group was composed of 76 physically and mentally healthy young subjects from the general population living in the same region. Controls’ average age ( ± S.D) was 27.1 ( ± 11.2) years, with a female predominance (M: 29.3%; F: 70.7%).

### HRQOL assessment tool

The short form 36 (SF-36) health survey is a generic HRQoL tool used in an extensive spectrum of medical conditions. This instrument relies upon patient self-reporting and is widely utilized for routine monitoring and assessment of care outcomes in adult patients. It is now freely available in English and different other languages and dialects (18). It measures physical and mental well-being and constructs eight health concepts:

1. Physical functioning (PF) (10 items)
2. Role-physical (RP) (4 items)
3. Bodily pain (BP) (2 items)
4. General health (GH) (5 items)
5. Vitality (VT) (4 items)
6. Social functioning (SF) (2 items)
7. Role-emotional (RE) (3 items)
8. Mental health (MH) (5 items)

A final item, termed “recent change in general health,” is answered by the patient but is not included in the scoring process. Scales are standardized with a complex scoring algorithm (19). Each health axis ranges from 0 to 100. Higher scores indicate better health status. Each domain scoring < 65 is considered altered. The development of online SF-36 calculators has facilitated its integration into everyday clinical practice (20). In the current survey, we used a validated version of the SF-36 translated into the Tunisian dialect (21).

### Ethical considerations

This study does not raise any ethical concerns. We provided clear information about the survey to all participants in order to obtain their written consent.

### Statistical analysis

We used descriptive statistics for the sociodemographic and clinicopathologic features of the subjects. Mean SF-36 domain scores were compared in subjects and controls. To investigate demographic, disease, and treatment-related risk factors that could influence each domain of HRQoL, we compared two subgroups of DTC survivors (with altered HRQoL versus without altered HRQoL). For the study of continuous variables, the Student’s t-test or the Mann-Whitney U-test were used, depending on the normality of the distribution. The chi-square test was chosen for nominative parameters. We analyzed the influence of TSH suppression and RAI cumulative dose on the HRQoL based on Spearman’s correlations. In all analyses, a p-value < 0.05 (two-tailed) was considered to be statistically significant. All analyses were performed using IBM SPSS Statistics (version 25.0).

## Results

### Patient recruitment

From September 1^st^ to November 30^th^, 2021, 266 patients diagnosed with DTC attended the outpatient clinic of the Department of Nuclear Medicine, Habib Bourguiba University Hospital, Sfax, Tunisia, for routine follow-up. Our recruitment procedure is explained in **Figure 1**.

We selected 201 consecutive patients who were potentially disease-free survivors of DTC. Sixty-seven subjects (33.3%) were excluded either because they were still undergoing RAI therapy, had a medical history of mental illness, suffered from a concomitant extrathyroidal cancer, or were illiterate.

Thirty-six of the 134 (26.9%) eligible DTC-free survivors declined participation. Overall, 98 patients (73.1%) were included in our survey. Twelve answers were counted out due to incomplete responses to the SF-36 questionnaire.

### Baseline Tunisian DTC survivors’ characteristics

The baseline clinical characteristics of the Tunisian participants are summarized in **Table 1**. At the time of the study, the average age ( ± SD) was 44.3 years ( ± 12.5). The DTC-free survival rate for these eligible patients was 5.8 years ( ± 4.8). Participants were predominantly female (89.5%). Most of the DTC survivors were blue-collar workers (87%). The majority had no medical history (65.1%). In 91.9% of the patients, papillary thyroid carcinoma was the most common histological type. The pathological findings featured multifocal carcinoma in 38.3% of the cases. DTCs were microcarcinomas in 34.9%. Half of DTC survivors had stage pT1 disease (55.8%). Cervical lymph node metastases were found in 52.3% of DTCs. Distant metastases were encountered in 6 patients (7%). Almost three-quarters of DTCs were in the ATA low-risk (34.9%) and intermediate-risk (43%) categories. All patients had undergone TT with pCNL and RAI remnant ablation. The mean dose of radioiodine 131 required to achieve remnant ablation was 274.4 (± 249.7) mCi. TSH suppression therapy was prescribed for all patients until achieving an excellent response. At the time of the survey, the average suppressed TSH level was 0.42 (± 0.86) mIU/L.

**Table 1:** Baseline characteristics of baseline Tunisian DTC survivors’ characteristics.

Tunisian DTC survivors'Characteristics	Variables
Total DTC survivors	N=86
Age at diagnosis (yr), mean (±SD)	38.5 (± 12.4)
Age at evaluation (yr), mean (±SD)	44.3 (± 12.5)
Survivorship (yr), mean (±SD)	5.8 (± 4.8)
Gender	
Male, n(%)	9 (10.5%)
Female, n(%)	77 (89.5%)
Menopausal women, n(%)	27 (34.6%)
Education	
Primary school degree, n(%)	31 (36%)
High school degree, n(%)	33 (38.4%)
University degree, n(%)	22 (25.6%)
Profession	
Unemployed, n(%)	17 (19.8%)
Blue-collar worker, n(%)	60 (69.8%)
White-collar worker, n(%)	9 (10.5%)
Nombre of comorbidities	
None, n(%)	56 (65.5%)
1, n(%)	14 (16.3%)
≥ 2, n(%)	16 (18.6%)
DTC pathology	
Papillary thyroid carcinoma, n(%)	79 (91.9%)
Follicular thyroid carcinoma, n(%)	7 (8.1%)
Unifocal DTC, n(%)	33 (38.3%)
Multifocal DTC, n(%)	43 (61.7%)
Microcarcionoma, n(%)	30 (34.9%)
T stage	
pT1, n(%)	48 (55.8%)
pT2, n(%)	15 (17.4%)
pT3, n(%)	19 (22.1%)
pT4, n(%)	2 (2.3%)
Unknown, n(%)	2 (2.3%)
Cervical lymph node metastases	
pN0, n(%)	45 (52.3%)
pN+, n(%)	41 (47.7%)
Distant Metastases	
M0, n(%)	80 (93%)
M+, n(%)	6 (7%)
ATA risk category	
Low-risk DTC, n(%)	30 (34.9%)
Intermediate-risk DTC, n(%)	37 (43%)
High-risk DTC, n(%)	19 (22.1%)
Management protocol	
Total thyroidectomy, n(%)	86 (100%)
pCNL , n(%)	86 (100%)
RAI remanant ablation, n(%)	86 (100%)
RAI cumulative dose (mCi), mean (±SD)	274.4 (± 249.7)
TSH suppression therapy, n(%)	86 (100%)
TSH level at evaluation (mIU/L), mean (±SD)	0.42 (± 0.86)

DTC, Differentiated thyroid cancer; ATA, American Thyroid Association; pCNL, prophylactic central neck lymphadenectomy; RAI, radioactive iodine 131; TSH, Thyroid-stimulating hormone.

### Comparison of SF-36 scores between DTC survivors and Tunisian healthy controls

**Figure 2:**
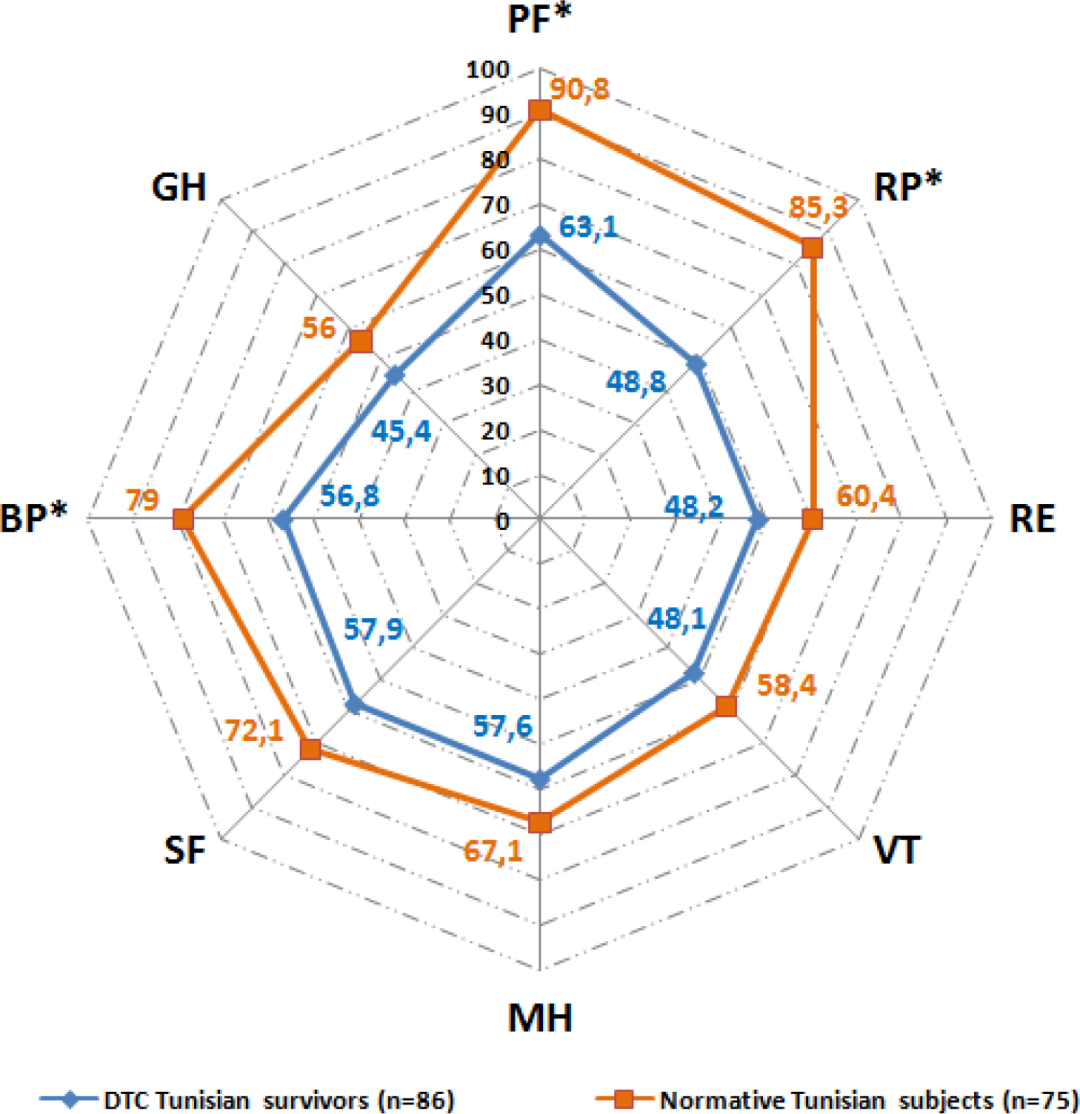
Comparison of average SF-36 scores between DTC survivors and healthy Tunisian controls. DTC, Differentiated thyroid cancer; SF-36, Short form 36 health survey; PF, Physical Functioning; RP, Role-Physical; RE, Role-Emotional; VT, Vitality; MH, Mental Health; SF, Social Functioning; BP, Bodily Pain; GH, General Health. * statistically significant alteration p<0.05 (Student’s t-test).

The comparison of results from the SF-36 scores per domain between DTC survivors and Tunisian controls is recapitulated in **Figure 2**. Tunisian patients with a history of DTC featured significantly lower scores in the domains related to physical health. Mean scores in the PF, RP, and BP domains were substantially decreased compared to the reference population. Although DTC survivors had lower scores in domains related to mental health (i.e., MH, RE, and VT) and social functioning, these differences were not statistically significant. Lower general health scores were frequently reported in the DTC survivors set side by side with healthy controls.

### Factors linked to altered HRQoL in Tunisian DTC survivors

In **Table 2**, we summarized the influence of different demographic, clinical, and therapeutic features of DTC on HRQoL. The correlations between SF-36 scores and continuous variables are compiled in **Table 3**.

**Table 2:** Sociodemographic, clinical, and therapeutic determiants of HRQoL alteration in Tunisian DTC survivors.

HRQOL determinants		Altered PF		Altered RP		Altered RE		Altered VT		Altered MH		Altered SF		Altered BP		Altered GH	
**Gender**	Female	86.4%	NS	89.1%	NS	90.9%	NS	91.4%	NS	91.2%	NS	90.9%	NS	94.2%	NS	89.2%	NS
	Male	13.6%		10.9%		9.1%		8.6 %		8.8%		9.1%		5.8%		10.8%	
**Menopause**	Yes	61.5%	NS	57.1%	NS	57.5%	NS	66.2%	NS	67.3%	NS	56.9%	p=0.038*	63.3%	NS	65,3%	NS
	No	38.5%		42.9%		42.5%		33.8%		32.7%		43.1%		36.7%			
**Profession**	Blue-collar	94.7%	p=0.029*	91.9%	NS	88.6%	NS	87.3%	NS	89.1%	NS	86.4%	NS	89.7%	NS	89,4%	p=0.000*
	White-collar	5.3%		8.1%		11.4%		12.7%		10.9%		13.6%		10.3%		10,6%	
**Comorbidities**	None	54.5%	NS	54.3%	p=0.040*	56.8%	NS	67.1%	NS	64.9%	NS	58.2%	NS	51.9%	NS	65,1%	NS
	≥1	45.5%		45.7%		43.2%		32.9%		35.1%		41.8%		49.1%		34,9%	
**Histology Findings**	PTC	90.9%	NS	89.1%	NS	90.9%	NS	91.4%	NS	89.5%	NS	90.9%	NS	92.3%	NS	92.8%	NS
	FTC	9.1%		10.9%		9.1%		8.6%		10.5%		9.1%		7.7%		7.2%	
**DTC Focality**	MFC	43.5%	NS	38.1%	NS	42.1%	NS	44.1%	NS	37.0%	NS	36.0%	NS	38.1%	NS	42.1%	NS
	UFC	56.5%		61.9%		57.9%		55.9%		63.0%		64.0%		61.9%		57.9%	
**T stage**	T1	50.0%	NS	58.7%	NS	56.8%	NS	57.4%	NS	59.6%	NS	58.2%	NS	62.0%	NS	59.3%	p=0.026*
	T2	25.0%		23.9%		27.3%		20.6%		15.8%		20.0%		20.0%		16.0%	
	T3	22.7%		15.2%		13.6%		19.1%		22.8%		20.0%		16.0%		22.2%	
	T4	2.3%		2.2%		2.3%		2.9%		1.8%		1.8%		2.0%		2.5%	
**Lymph node metastases**	N0	36.4%	p=0.006*	45.7%	NS	50.0%	NS	52.9%	NS	54.4%	NS	52.7%	NS	56.0%	NS	50.6%	NS
	N+	63.6%		54.3%		50.0%		47.1%		45.6%		47.3%		44.0%		49.4%	
**Distant metastases**	M0	93.2%	NS	95.7%	NS	95.5%	NS	92.9	NS	94.7%	NS	96.4%	NS	96.2%	NS	92.8%	NS
	M+	6.8%		4.3%		4.5%		7.1%		5.3%		3.6%		3.8%		7.2%	
**ATA risk classification**	Low	27.3%	NS	34.8%	NS	38.6%	NS	34.3%	NS	42.1%	NS	40.0%	NS	38.5%	NS	36.1%	NS
	Intermediate	45.5%		45.7%		40.9%		45.7%		36.8%		40.0%		44.2%		41.0%	
	High	27.3%		19.6%		20.5%		20.0%		21.1%		20.0%		17.3%		22.9%	
**Low quality scar healing**	No	93.2%	NS	91.3%	NS	86.4%	NS	91.4%	NS	87.7%	p=0.049*	89.1%	NS	88.5%	NS	91.6%	NS
	Yes	6.8%		8.7%		13.6%		8.6%		12.3%		10.9%		11.5%		8.4%	
**Post-operative hypoparathyroidism**	No	70.5%	NS	76.1%	NS	72.7%	NS	78.6%	NS	84.2%	p=0.038*	81.8%	NS	82.7%	NS	79.5%	p=0.008*
	Yes	29.5%		23.9%		27.3%		21.4%		15.8%		18.2%		17.3%		20.5%	
**Recurrent laryngeal nerves injury**	Yes	58.8%	NS	58.8%	NS	64.7%	NS	88.2%	NS	64.7%	NS	64.7%	NS	47.1%	NS	94.1%	NS
	No	50.0%		52.9%		48.5%		80.9%		67.6%		64.7%		64.7%		98.5%	
**RAI-induced sialadenitis**	No	75.0%	NS	73.9%	NS	75.0%	NS	78.6%	NS	73.7%	NS	76.4%	NS	76.9%	NS	78.3%	NS
	Yes	25.0%		26.1%		25.0%		21.4%		26.3%		23.6%		23.1%		21.7%	
**Subclinical thyroitoxicosis**	No	34.1%	p=0.003*	37,0%	p=0.013*	40.9%	NS	45.7%	NS	45.6%	NS	43.6%	NS	46.2%	NS	48.2%	NS
	Yes	65.9%		63.0%		59.1%		54.3%		54.4%		56.4%		53.8%		51.8%	
**palpitations**	No	56.8%	p=0.037*	65.2%	NS	68.2%	NS	62.9%	NS	70.2%	NS	69.1%	NS	65.4%	NS	66.3%	NS
	Yes	43.2%		34.8%		31.8%		37.1%		29.8%		30.9%		34.6%		33.7%	
**Asthenia**	non	29.5%	NS	21.7%	p=0.017*	22.7%	p=0.038*	30.0%	NS	31.6%	NS	29.1%	NS	26.9%	NS	31.3%	p=0.041*
	Yes	70.5%		78.3%		77.3%		70.0%		68.4%		70.9%		73.1%		68.7%	
**Irritability**	No	38.6%	p=0.006*	39.1%	p=0.006*	47.7%	NS	51.4%	NS	45.6%	p=0.048*	45.5%	p=0.049*	53.8%	NS	53.0%	NS
	Yes	61.4%		60.9%		52.3%		48.6%		54.4%		54.5%		46.2%		47.0%	
**Insomnia**	No	44.2%	p=0.033*	42.2%	p=0.008*	41.9%	p=0.010*	51.5%	NS	50.0%	NS	49.1%	NS	51.0%	NS	56.8%	NS
	Yes	55.8%		57.8%		58.1%		48.5%		50.0%		50.9%		49.0%		43.2%	
**Weight loss**	No	86.4%	NS	87.0%	NS	88.6%	NS	90.0%	NS	89.5%	NS	90.9%	NS	90.4%	NS	89.2%	NS
	Yes	13.6%		13.0%		11.4%		10.0%		10.5%		9.1%		9.6%		10.8%	
**Heat intolerance**	No	77.3%	NS	82.6%	NS	81.8%	NS	81.4%	NS	80.7%	NS	81.8%	NS	88.5%	NS	84.3%	NS
	Yes	22.7%		17.4%		18,2%		18.6%		19.3%		18.2%		11.5%		15.7%	
**Fear of cancer recurrence**	No	45.2%	NS	40.9%	NS	41.9%	NS	43.3%	NS	44.4%	NS	39.6%	NS	38.0%	NS	41.8%	NS
	Yes	54.8%		59.1%		58.1%		56.7%		55.6%		60.4%		62.0%		58.2%	

HRQoL, Health-related quality of life ; DTC, Differenciated thyroid cancer ; PF, Physical Functioning; RP, Role-Physical; RE, Role-Emotional; VT, Vitality; MH, Mental Health; SF, Social Functioning; BP, Bodily Pain; GH, General Health; PTC, papillary thyroid cancer ; FTC, Follicular thyroid cancer ; UFC, Unifocal thyroid cancer ; MFC, Multifocal thyroid cancer ; ATA, American Thyroid Association ; RAI, Radioactive iodine 131.

*statistically significant difference p<0.05 (Chi-square test). The bold values represent the statistically significant results.

**Table 3:** Spearman’s correlations between SF-36 scores and clinical and therapeutic features of DTC in Tunisian survivors.

SF-36 domains	PF	RP	RE	VT	MH	SF	BP	GH
Age at diagnosis	-0.219^*^	-0.351^*^	-0.259^*^	-0.092	-0.155	-0.244^*^	-0.216^*^	-0.033
Age at evaluation	-0.303^*^	-0.327^*^	-0.245^*^	-0.055	-0.028	-0.214^*^	-0.094	-0.073
DTC survivorship	-0.133	-0.013	-0.033	0.015	0.217^*^	-0.065	0.181	-0.092
RAI cumulative dose	-0.136	-0.028	0.010	0.137	0.174	0.146	0.218^*^	-0.022
Suppressed TSH at evalution	-0.133	-0.168	-0.197	-0.212	-0.364^*^	-0.280^*^	-0.229	-0.134

DTC, Differenciated thyroid cancer; SF-36, Short Form 36 health survey; PF, Physical Functioning; RP, Role-Physical; RE, Role-Emotional; VT, Vitality; MH, Mental Health; SF, Social Functioning; BP, Bodily Pain; GH, General Health; RAI, Radioactive iodine 131; TSH, Thyroid-stimulating hormone.

*statistically significant correlation p<0.05.The bold values represent the statistically significant results.

#### a. Social and demographic backgrounds

Our results highlight a significant correlation between age and most of the SF-36 physical and emotional aspects. Age was negatively associated with PF (r = -0.3; p = 0.005), RP (r = -0.32; p = 0.002), RE (r = -0.24; p = 0.024), and SF (-0.21; p = 0.049). Gender did not significantly influence any HRQoL domain. SF was significantly altered in menopausal female DTC survivors (p = 0.038). Among the DTC survivors, blue-collar workers had significantly lower PF (p = 0.029) and GH (p = 0.000) in comparison with the white-collar category.

#### b. Clinical and pathological Tunisian DTC peculiarities

Having one or more additional somatic comorbidities besides DTC was significantly linked to altered RP scores (p = 0.04). Neither the histological pattern (papillary or follicular) nor the multifocality of DTC influenced SF-36 domain scores. Tumor size was linked to altered GH (p = 0.026). Patients with lymph node metastases (N+) at diagnosis had significantly lower PF scores (p = 0.006). Our results did not establish any remarkable relationship between the presence of initial distant metastases and altered SF-36 scores. Patients belonging to ATA intermediate or high-risk subgroups displayed altered scores in PF, RP, VT, and BP even though these tendencies were not significant. The cancer-free survivorship duration was positively correlated with the MH score (r = 0.21; p = 0.046).

#### c. DTC management protocol

TT and pCNL are the conventional surgical procedures in our center. Surgical-related morbidity occurred in 33.7% of cases. Post-operative hypoparathyroidism (pHPT) and recurrent laryngeal nerve injury were the most common complications, reported in 23.3% and 20.9% of patients, respectively. Poor neck scar healing was observed in 8.1% of patients. The interviewed DTC survivors did not mention any other long-term post-operative complications. Lower MH scores were observed in patients with poor quality neck scar healing (p = 0.049) and those with persistent pHPT (p = 0.038). Recurrent laryngeal nerve injury did not affect any SF-36 domain in our sample.

Isotopic remnant ablation is the second-line management option for DTC. It is usually performed 3-6 months after surgery. RAI complications were encountered in 26.7% of patients, mainly sialadenitis (22.1%). None of the RAI-related complications had a significant influence on HRQoL scores in our population. The BP score was positively correlated with the radioiodine cumulative dose (r = 0.21; p = 0.045).

The third pillar of management of DCT is long-term TSH suppression therapy, which may cause discomfort in several patients due to possible iatrogenic hyperthyroidism. Clinical complaints related to hyperthyroidism were noted in 51.2% of DTC survivors and were responsible for significantly reduced scores in PF (p = 0.003) and RP (p = 0.013). Some patients did exhibit palpitations, which affected mainly the physical aspect of HRQoL. Asthenia influenced negatively not only RP (p = 0.017) but also emotional aspects such as RE (p = 0.034) and GH (p = 0.041). Patients suffering from irritability had significantly impacted scores in PF (p = 0.033), RP (p = 0.008), and RE (p = 0.01). TSH suppression was negatively and notably correlated with MH (r = -0.36; p = 0.000) and SF (r = -0.28; p = 0.024) scores.

As for the fear of cancer recurrence, it did not influence the HRQoL in our survey.

## Discussion

Whether DTC-free patients, in Tunisia and in other southern Mediterranean and African countries, display impaired HRQoL is an overlooked subject. To address this matter and to determine the major determinants of HRQoL in this population, we conducted this cross-sectional comparative study. Our work uncovered interesting findings.

Despite being disease-free, DTC survivors exhibited deteriorated aspects of HRQoL in comparison with healthy controls who share an identical socio-economic and ethnic background. In our study, the SF-36 items that reflect physical performance, such as GH, PF, RP, and BP, are the most adversely affected in DTC-cured subjects. Congruent results were found in most studies focusing on DTC survivors. Tan et al. mailed a self-administered questionnaire containing SF-36 to 290 consecutive patients treated for DTC. There was a statistically significant alteration in SF-36 scores between DTC survivors and a normative Singaporean population in all domains except for social functioning (SF) (22). Similarly, Lee et al. interviewed, in their cross-sectional survey, 316 disease-free DTC survivors and assessed their HRQoL by means of the ECORTC-QOQC30. They found a decreased HRQoL for DTC survivors in physical, role, cognitive, emotional, and social domains compared to an age-matched healthy control group (23).

Another finding of our study is that lower HRQoL scores were influenced by the sociodemographic background. Advanced age and the blue-collar category are associated with reduced physical HRQoL in DTC survivors. Menopause affects mostly the social functioning of female patients. Hussonet al. investigated the influence of sociodemographic peculiarities on the long-term HRQoL in DTC patients based on a large sample (n = 306) from the Eindhoven Cancer Registry (1990–2008). The authors concluded that long-term thyroid cancer survivors experience more symptoms and deteriorated HRQoL compared to age-and sex-matched cancer-free controls (n = 800) (24). This same study did emphasize that having one or more comorbidities was significantly associated with poorer global health, physical role, and emotional functioning as well as more fatigue (OR = 0.24) and pain (OR = 0.25), which supports our findings of impaired HRQoL in patients with a cured DTC who had comorbidities.

Except for the histological subtype, the pathology findings, i.e., larger tumor size and the presence of lymph node metastases, are disturbing factors for HRQoL. Interestingly, our results did not show a substantial effect of the presence of distant metastases or the ATA risk category on the HRQoL in this population.

Additionally, we identified longer survivorship as an improving determinant of the psychological aspect of HRQoL in disease-free DTC individuals. Conflicting conclusions were reported in some previous papers. For example, in a prospective Swedish population-based study including 279 long-term DTC survivors, distress about disease recurrence a decade after diagnosis continues to negatively affect HRQoL in DTC-cured patients, with an impact on HRQoL comparable to actual recurrence (25).

Thyroid surgery and post-operative complications represent serious determinants of HRQoL. Our center’s surgical strategy, consisting of systematic TT plus pCNL, could prevent disease recurrence, but it is associated with a high morbidity incidence compared with similar interventions in specialized European centers (26). This aggressive surgical procedure seems to compromise mental well-being in recruited DTC survivors since neck dissection is associated with greater morbidity even in experienced hands (27). In a randomized prospective survey, Lee et al. compared two groups: a TT-only group (n = 104) and a TT plus pCNL group (n = 153). Postoperative complications were significantly more prevalent when pCNL was performed (28).

Recent publications have found that patients who had a TT are 1.5 times more likely to report an HRQoL issue or an adverse effect of treatment compared with patients who underwent a hemithyroidectomy (29). This alteration in HRQoL is more notable in patients undergoing both TT and pCNL. An Australian study conducted by Gane et al. assessed the outcomes of neck dissection within 5 years in head and neck cancer survivors using the Neck Dissection Impairment Index, a region-specific measure of HRQoL. Sixty-eight percent of the included patients demonstrated reduced HRQoL (30).

Our findings support that patients with persistent pHPT had significantly lower MH scores. Likewise, Büttner et al. divided 89 DTC patients into two groups based on the presence of pHPT. Patients in the pHPT group reported significantly reduced HRQoL in nine of the 15 scales of the EORTC QLQ-C30 compared to patients without pHPT. Regression analysis showed that pHPT was independently negatively associated with various scales of the QLQ-C30 (31).

Although no significant influence between recurrent laryngeal nerve injury and HRQoL was established in Tunisian DTC survivors, some publications highlighted a more deteriorated HRQoL due to voice impairment and dysphonia. In the large online survey of Goswamiet al. involving 1,743 thyroid cancer survivors from the United States, postoperative dysphonia (ß 1.83–3.07) predicted worse HRQoL scores (32).

Exposed neck surgical scars following thyroidectomies plus lymph node dissection, especially those with low-quality healing, represent a cosmetic issue that disturbs the patients’ psychological balance. Choi et al. used the Dermatology Life Quality Index (DLQI) to evaluate the impact of post-thyroidectomy scars on the quality of life of 79 thyroid cancer patients. Domain 2, related to daily activities, including clothing, was the most greatly impacted among patients. Interestingly, the HRQoL does not seem to be associated with the severity or type of the scar, but rather with the presence of the scar itself (33).

Our results dismiss any influence of RAI remnant ablation on HRQoL. However, greater cumulative doses of radioiodine were found to aggravate pain perception in DTC survivors. Taïeb et al. assessed the determinants of medium-term HRQoL after the initial RAI therapy in 83 DTC patients performing the validated Functional Assessment of Chronic Illness & Therapy (FACIT) at the time of inclusion (t0) and later at the 9-month post-RAI (t1). The prospective analysis confirmed that medium-term FACIT scores were not statistically different between t0 and t1 patients (34).

A Brazilian tertiary center cross-sectional study using the University of Washington Quality of Life questionnaire reported conflicting findings as postoperative radioactive iodine treatment was an important predictor of HRQoL in patients with thyroid cancer, affecting the domains of chewing, speech, taste, saliva, and anxiety. This study also concluded that patients who received higher doses (more than 150 mCi) of radioactive iodine are at risk for poor HRQoL (35).

Aiming to reduce the risk of recurrence, TSH suppression with supraphysiologic doses of levothyroxine is commonly used to treat patients with DTC. The ATA recommends lowering the initial TSH level to less than 0.1 mIU/L for high-risk DTC patients and 0.1-0.5 mIU/L for intermediate-risk patients. TSH may be maintained at the lower end of the reference range (0.5–2mIU/L) in low-risk subjects who have undergone successful remnant ablation and have undetectable serum thyroglobulin levels (5).

Adverse effects of TSH suppression may include several symptoms of subclinical thyrotoxicosis and are often observed in DTC survivors. Our results highlight that HRQoL is severely altered in patients experiencing treatment-induced asthenia and palpitations. The TSH suppression procedure has a negative impact on both physical and emotional well-being. Our data established a negative correlation between a suppressed TSH level and reduced psychological well-being and social functioning in the study population. Giusti et al. administered the Billewicz scale to 123 DTC patients. In comparison to 192 control subjects who had undergone surgery for benign thyroid pathology, DTC patients featured significantly higher scores on the hyperthyroid symptoms scale (36).

Some authors have hypothesized that a high level of free T3 is associated with altered physical performance and fatigue in DTC survivors undergoing TSH suppression. They suggest considering thyroid functional status, particularly the level of free T3, to alleviate the burden on HRQoL (37). The recent trend of individualizing treatment and avoiding aggressive interventions in low-risk cases of thyroid cancer is expected to have a positive impact on the HRQoL of long-term survivors (38).

Lastly, the fear of thyroid cancer recurrence did not influence the HRQoL in our sample. Our center policy is to provide clear information about the excellent prognosis and the treatment procedure of DTC to all referred patients. Poor physical and emotional functioning were significantly associated with bad ratings of information quality, more barriers to obtaining information, and a greater need for information among a sample of newly diagnosed head and neck cancer patients in a British study (39). A tailored message about DTC management and prognosis may attenuate cancer-induced anxiety and depression while maintaining HRQoL in DTC survivors (40).

The high response rate of 73.1% is one of the strengths of our survey, permitting a representative data collection of DTC survivors in the Tunisian population. The standard management (TT plus pCNL, RAI remnant ablation, and long-term TSH suppression) provided to all participants allows the formation of a homogeneous DTC patient sample, reducing treatment-related biases affecting HRQoL.

Our research still has some limitations. The SF-36 is a generic scale that provides a reliable assessment of physical, psychological, and social aspects of HRQoL in DTC survivors, but it does not take into consideration the spiritual well-being domain. Some cancer-specific HRQoL questionnaires such as THYCA-QoL and EORTC QLQ-C30 could have yielded more rigorous data; however, and to the best of our knowledge, there are no valid versions in Standard Arabic or Tunisian dialect of these questionnaires. Similarly, we could not perform additional comparative analyses because no baseline SF-36 values were available and information on HRQoL before surgery was missing. Eventually, due to the cross-sectional design and the short period of the study, temporal relations between risk factors and alteration of HRQoL could not be investigated.

In conclusion, HRQoL is substantially reduced in DTC survivors compared to the normative Tunisian population. These results could be scrupulously extrapolated to similar individuals who originated from the same South Mediterranean and African areas. This alteration is attributable to various sociodemographic, clinical, and iatrogenic factors that negatively impact the physical and mental health of these subjects. The survivorship plan of DTC in developing South Mediterranean and African countries should no longer be restricted to oncological-related outcomes. Long-term survivors also perceive their illness subjectively, socially, and emotionally. Therefore, a larger multidisciplinary approach is needed to improve their HRQoL and preserve their social and psychological well-being. The development of effective and coordinated aftercare interventions in this region is warranted to address the needs of the DTC survivor community, with a special focus on personalized information provision and coping skills.

## Data availability statement

The original contributions presented in the study are included in the article/supplementary material. Further inquiries can be directed to the corresponding author.

## Ethics statement

Ethical review and approval was not required for the study on human participants in accordance with the local legislation and institutional requirements. The patients/participants provided their written informed consent to participate in this study.

## Author contributions

Conception or design: AM, FH, and MM. Redaction of the manuscript: AM, and FH. Acquisition, analysis, or interpretation of data: AM, HC, and WG. Final approval of the manuscript: MA and FG. All authors contributed to the article and approved the submitted version.

## Acknowledgments

We thank Dr. Oumeyma Trimeche for her assistance with data interpretation and for her comments that greatly improved the manuscript.

## Conflict of interest

The authors declare that the research was conducted in the absence of any commercial or financial relationships that could be construed as a potential conflict of interest.

## Publisher’s note

All claims expressed in this article are solely those of the authors and do not necessarily represent those of their affiliated organizations, or those of the publisher, the editors and the reviewers. Any product that may be evaluated in this article, or claim that may be made by its manufacturer, is not guaranteed or endorsed by the publisher.
